# Structural signatures of thermal adaptation of bacterial ribosomal RNA, transfer RNA, and messenger RNA

**DOI:** 10.1371/journal.pone.0184722

**Published:** 2017-09-14

**Authors:** Clara Jegousse, Yuedong Yang, Jian Zhan, Jihua Wang, Yaoqi Zhou

**Affiliations:** 1 UFR Sciences et Techniques, Université de Nantes, 2 rue de la Houssinière, Nantes, France; 2 Institute for Glycomics and School of Information and Communication Technology, Griffith University, Gold Coast, QLD, Australia; 3 Shandong Provincial Key Laboratory of Biophysics, Institute of Biophysics, Dezhou University, Dezhou, China; Max-Planck-Institut fur terrestrische Mikrobiologie, GERMANY

## Abstract

Temperature adaptation of bacterial RNAs is a subject of both fundamental and practical interest because it will allow a better understanding of molecular mechanism of RNA folding with potential industrial application of functional thermophilic or psychrophilic RNAs. Here, we performed a comprehensive study of rRNA, tRNA, and mRNA of more than 200 bacterial species with optimal growth temperatures (OGT) ranging from 4°C to 95°C. We investigated temperature adaptation at primary, secondary and tertiary structure levels. We showed that unlike mRNA, tRNA and rRNA were optimized for their structures at compositional levels with significant tertiary structural features even for their corresponding randomly permutated sequences. tRNA and rRNA are more exposed to solvent but remain structured for hyperthermophiles with nearly OGT-independent fluctuation of solvent accessible surface area within a single RNA chain. mRNA in hyperthermophiles is essentially the same as random sequences without tertiary structures although many mRNA in mesophiles and psychrophiles have well-defined tertiary structures based on their low overall solvent exposure with clear separation of deeply buried from partly exposed bases as in tRNA and rRNA. These results provide new insight into temperature adaptation of different RNAs.

## Introduction

Since bacteria first appeared on the Earth several billion years ago, they have colonized every part of the planet ranging from frigid-cold polar regions and stratospheres to super-hot hydrothermal vents. Bacteria adapted to different temperatures were classified into psychrophiles (<24°C), mesophiles (24°C-50°C), thermophiles (50°C-80°C), and hyperthermophiles (>80°C) according to their optimal growth temperatures (OGT). Temperature adaptation of bacterial biomolecules is a subject not only of fundamental interest in molecular evolution and adaptation [1] but also of practical interest of biotech industries [2–4]. Temperature adaptation requires coordinated changes in all biologically active molecules [5], at the genome (DNA), transcriptome (RNA), and proteome (proteins) levels, in particular.

Temperature adaptation of proteins has been a subject of intensive studies for several decades [6–9]. These studies revealed that thermophilic proteins were stabilized by multiple factors including deletion of surface loops [10, 11], tight packing by more branched hydrophobic side chains [12–15], and increased use of salt bridge network [16–18]. Thus, temperature adaptation of proteins occurs at both sequential and three-dimensional structural levels.

At the DNA level, most studies were limited to analysis of the composition of four nucleotides. The binding affinity of a double stranded-DNA is strongly depending on its nucleotide composition because the base pair between guanine (G) and cytosine (C) is bound by three hydrogen bonds, compared to two hydrogen bonds between adenine (A) and thymine (T). Thus, one might expect that the genomes of thermophilic species have higher GC content, relative to mesophiles and psychrophiles, in order to counteract thermal denaturation. Opposite to the expectation, there is no correlation between the GC content of the genome and the OGT of bacteria [19–21]. DNA was found stabilized by other techniques such as increase of ionic strength, cationic proteins, and supercoiling [22, 23].

At the RNA level, higher GC contents were observed in thermophilic ribosomal RNA (rRNA) [19, 24–26], transfer RNA (tRNA) [19, 24] and functional noncoding RNA [27], but not in messenger RNA (mRNA) [19, 24, 28]. For mRNA, an increased frequency of purine (G+A) was observed [29, 30]. Codon usage in mRNA of different species was also shown to be different at different OGT [29, 31] but their link to temperature adaption is not clearly established [32].

In contrast to compositional analysis of RNA bases, very little is known about temperature adaptation of RNA structures. Dutta and Chaudhuri showed that the secondary structure of tRNA was more stably folded [33]. Mallik and Kundu found that the tertiary structure of thermophilic 16S rRNA is more packed than that of mesophilic one [34]. Limited knowledge is largely due to the fact that RNA structures are challenging to determine experimentally while computational prediction of tertiary structure is far from accurate [35]. Moreover, *ab initio* prediction of tertiary structure [36–38] predicts the structures of isolated RNA chains that may or may not reflect their functional conformations *in vivo*.

Recently, we have developed a method called RNAsnap that makes sequence-based prediction of solvent accessible surface area (ASA) of RNA bases in its tertiary structure [39]. The method, that was trained by using protein-bound RNA structures, achieved with correlation coefficients (*r*) above 0.6 between predicted and actual solvent accessible surface area for five fold cross validation and independent test. However, it has a poor performance for protein-free RNA structures (*r*~0.2). Similarly, it correlates strongly with the accessibility of 6178 human mRNA sequences to dimethyl sulfate (DMS) experimentally measured in *in vivo* (*r* = 0.37) [40] but not *in vitro* (*r* = 0.07). Although only unpaired, exposed adenine and cytosine residues were detected by DMS, it was used successfully to approximate solvent accessibility [40]. These results suggest that RNAsnap can predict ASA of functional structures of RNAs without the information from their interacting partners. Bound structures are likely formed through conformational selection upon binding, as supported by experimental evidence [41, 42].

In this study, we will apply RNAsnap along with secondary structure prediction by RNAfold [43] to investigate the role of secondary and tertiary structure in temperature adaptation of bacterial rRNA, tRNA, and mRNA. RNAsnap was built based on X-ray structures crystallized at low temperatures. In other words, it is not feasible to determine ASA as a function of temperature. Thus, our investigation of temperature adaptation focused on how RNA sequences encode secondary structure and solvent accessible surface area differently for species with different OGTs. This is a generally accepted practice as there is simple no other alternative to analyze temperature-dependent behavior. For example, thermophilic and mesophilic 16S rRNAs were compared directly, despite that they were crystalized at the same temperature of 277K [34]. Here, we showed that rRNA and tRNA have very different temperature adaptation from mRNA. rRNA and tRNA in thermophiles and hyperthermophiles retain their structures whereas the corresponding mRNA behaves like random sequences without significant secondary or tertiary structures.

## Materials and methods

### Datasets

Three datasets of RNA sequences were built from the bacterial species with known OGTs. We obtained 729 prokaryote species with OGTs compiled by Lobry and Necsulea [32] and 131 extremophile species with OGTs available from the BacDive metadatabase [44]. The scientific names of these species were mapped to NCBI’s taxon identifiers (taxids) [45]. After limiting to bacterial species and only one strain per species, we obtained 536 retrieved taxids to search against the Reference Sequence (RefSeq) database [46] for well annotated bacterial genomes. There are 5,507 sequences of 20 tRNA coding genes from 289 species, and 9,624 mRNA sequences from 172 essential protein-coding genes [47] in 287 species, and 107 5S rRNA sequences from 107 species (not all the species have the same genes annotated). Here, we chose 5S rRNA to represent rRNA because 16S and 23S rRNAs were annotated in less than 20 bacterial species. However, the number of 5S RNA sequences is still much smaller than those of mRNA and tRNA. To increase the statistics of 5S rRNA, additional sequences were retrieved manually from the NCBI nucleotide database using the scientific names of bacteria species with available OGT in the BacDive metadatabase. The final set has 158 sequences of 5S rRNA from 158 species.

In addition to natural sequences, we also generated random RNA sequences with the same dinucleotide frequencies. Using dinucleotide frequencies, rather than mononucleotide frequencies, for generating random sequences is necessary because RNA secondary structure depends on pairwise stacking energies [48]. Using Ushuffle [49], we randomly shuffled dinucleotide within each original RNA sequence to obtain the corresponding random RNA sequence. These random RNA sequences have the same length, GC content and other dinucleotide frequencies as their original RNA sequences. Only one random sequence was generated per RNA chain as the main purpose is to demonstrate the ability of RNAfold and RNAsnap to distinguish natural sequences from random sequences, which presumably do not fold into well-defined structures.

### RNA secondary and solvent accessibility prediction

We downloaded and installed RNAfold from the ViennaRNA Package 2 [43]. RNAfold predicts the minimum free energy (MFE) of a single RNA sequence using the algorithm of Zuker and Stiegler [50] and calculates equilibrium base pairing probabilities using the partition function [51]. The base pairing probabilities are employed to obtain the percentage of paired nucleotides. All default parameters were employed.

The solvent accessible surface area (ASA) of RNA was predicted by the online server of the RNA SolveNt Accessibility Prediction (RNAsnap) at http://sparks-lab.org [39].

### Experimentally determined ASA values

The structures of *Escherichia coli*’s lysine-tRNA, a segment of mRNA and *Thermus thermophilus’s* 5S rRNA were extracted from the structure of *Thermus thermophilus* 70S ribosome in complex with the mRNA segment, tRNA^fMet^ and near-cognate tRNA^Lys^ (PDB 5IB8) [52]. The ASA of each nucleotide in tRNA, mRNA, and rRNA structures was calculated by PyMOL.

### Data average

To reveal the trend, all quantities of RNA chains (the GC contents, predicted percentage of paired nucleotides, predicted chain-average ASA, and standard deviation of predicted ASA values in a chain) are averaged over the species with the same OGT (i.e., species are clustered by bins of one Celsius degree in OGT). Statistical significances (p-value) between OGT dependences were calculated based on the average values, rather than the data from each species to avoid bias toward temperatures with many species. This is because we are only interested in the difference in trends of OGT dependences.

## Results

### Primary structure in temperature adaptation

**Fig 1:**
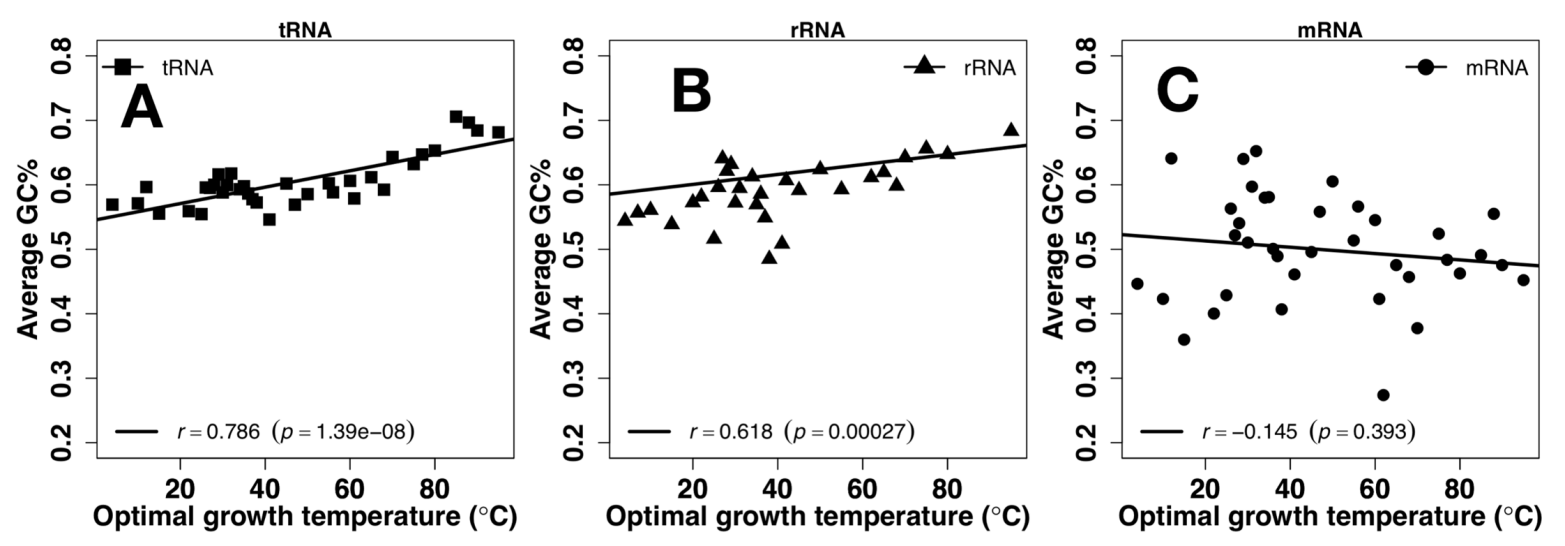
Primary structure vs. Temperature. Average GC content as a function of optimal growth temperature for tRNA (A), rRNA (B), and mRNA (C).

Because GC contents are commonly employed to investigate temperature adaptation, we examined the averaged GC contents at each Celsius degree as a function of OGT. As shown in Fig 1, there are strong positive correlations for tRNA (*r* = 0.786, p = 1.39e-08) (Fig 1A) and for rRNA (*r* = 0.618, p = 0.00027, Fig 1B) but not for mRNA (*r* = -0.145, p = 0.393, Fig 1C).

### Secondary structure in temperature adaptation

**Fig 2:**
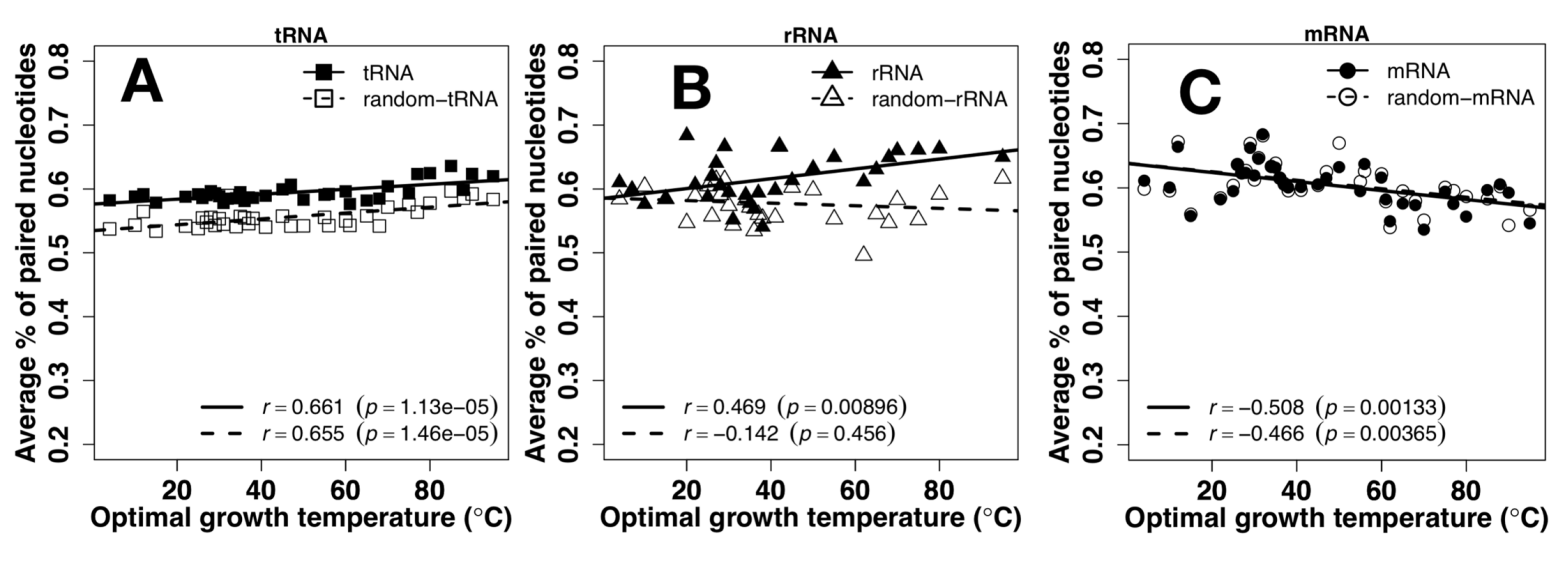
Secondary structure vs. temperature. Average number of paired nucleotides predicted by RNAfold as a function of optimal growth temperature for tRNA (A), rRNA (B), and mRNA (C). Results from random sequences are shown in open symbols.

Fig 2 examines the overall trend of secondary-structure based on the average fraction of predicted paired nucleotides of RNAs as a function of OGT of their corresponding species. The results from actual RNA sequences (represented by filled circles) are compared to those of random RNA sequences (represented by open circles). For tRNA (Fig 2A), strong positive correlations (p<0.001) are observed between secondary structure fractions and OGT for both actual and random sequences with similar, nearly flat slope, suggesting that the OGT-dependent increase in secondary structure is largely due to increase in GC contents of tRNA. The increment of paired nucleotides from low to high OGT, however, is only between 57.6% and 63.5% for natural sequences and between 53.4% and 59.7% for random sequences. That is, the increment is mainly due to the change of nucleotide composition in response to OGT changes. On the other hand, there is an increase in secondary structure for natural sequences of rRNA for temperature adaptation (*r* = 0.469, p = 0.00896) but not for random sequences (*r* = -0.142, p = 0.456) (Fig 2B). For mRNA (Fig 2C), a negative correlation is observed for both actual and random sequences (*r* = -0.580, -0.466 and p = 0.00133, 0.00365, respectively). That is, there is a loss of secondary structure and this loss is due to changes in compositions because the difference between natural and random sequences is not significant (p = 0.35). Thus, nucleotide compositions were the dominant factor in different temperature adaptation of secondary structure contents for tRNA (increase) and mRNA (decrease) whereas rRNA sequences were optimized for increasing in secondary structure content at higher OGT.

### Illustration of predicted and actual ASA values for tRNA and rRNA

Before we apply RNAsnap to monitor the relation between OGT and ASA, it is necessary to get a sense of the performance of RNAsnap in predicting ASA by using illustrative examples. We employed *Escherichia coli*’s lysine-tRNA and *Thermus thermophilus* 5S rRNA, both from PDB 5IB8. This structure was deposited in February 22, 2016 and released in May 25, 2016 [52]. We found that the newly deposited tRNA and rRNA sequences are not in sequence-homologous relation to any RNA chains employed in the training RNAsnap [39] based on sequence similarity determined by the software CD-HIT-est[53]. Thus, it can be considered an independent test example for RNAsnap.

**Fig 3:**
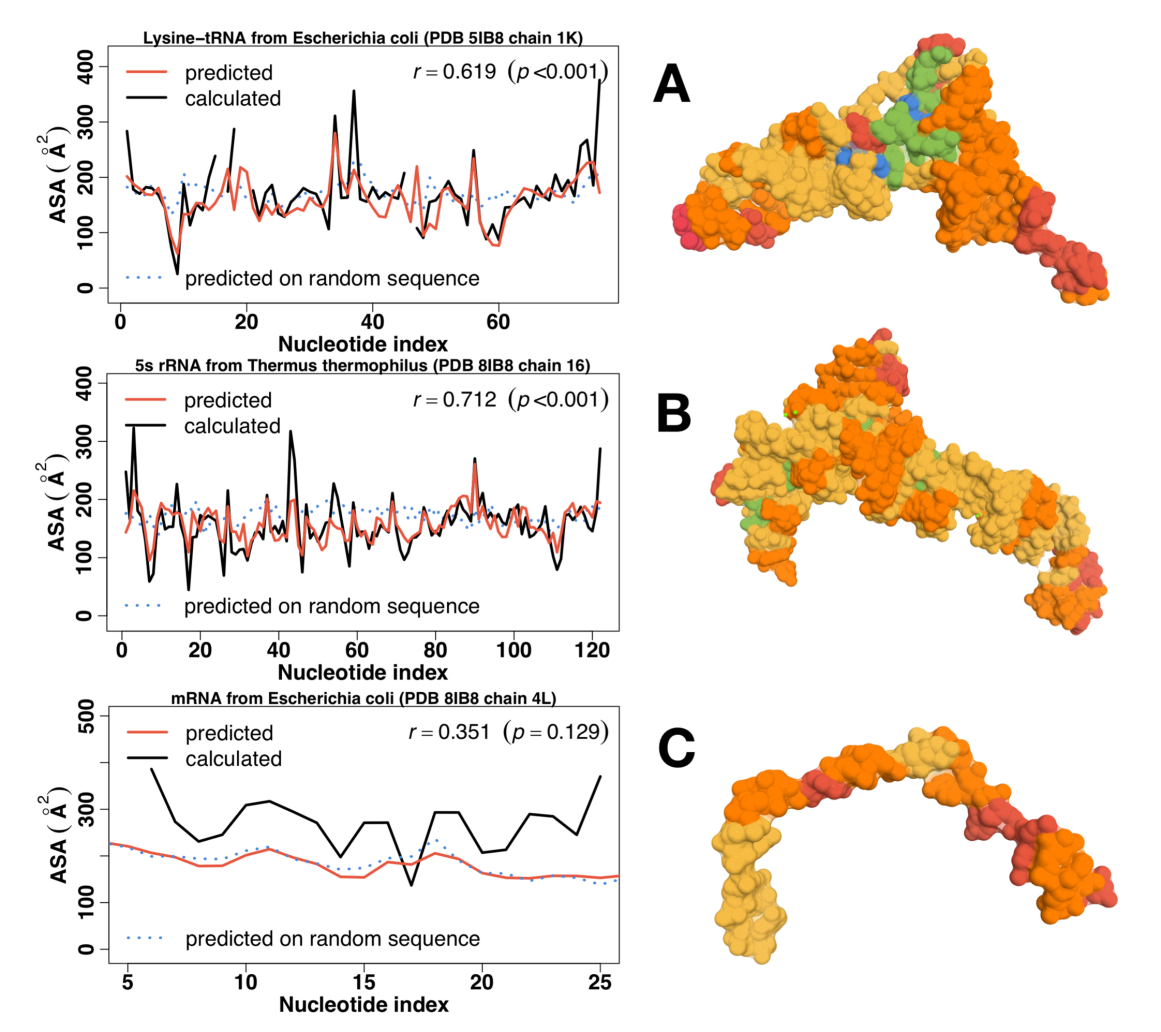
Predicted (Red) versus actual (Black) solvent accessible surface area (ASA) of an RNA along with its structure colored coded for accessibility. Results of a corresponding random sequence (randomly shuffled from the original sequence) were shown in Blue. (A) Lysine-tRNA from *E*. *coli* (PDB: 5IB8, chain: 1K), (B) 5S RNA from *Thermus thermophilus* (PDB: 5IB8, chain: 16) and (C) *E coli’s* mRNA in the same complex (PDB: 5IB8, chain: 4L) Experimental structures were color-coded according to the relative ASA: 5+ for Red, 4 for Orange, 3 for Yellow, 2 for Green and 1 for Blue.

Fig 3A compares predicted with calculated actual ASA values (in Å2) of *Escherichia coli*’s lysine-tRNA as a function of residue indices. It is clear that predicted ASA values follow the similar variations as actual ASA values with a Pearson correlation coefficient (*r*) of 0.619 between them (*p = 6*.*899e-09*). Fig 3A further projected predicted relative ASA of each nucleotide onto the tRNA structure by using the color scale defined according to predicted ASA. The figure confirmed that predicted buried regions are in the actual structural core region of tRNA. Similar results were observed for *Thermus thermophilus* 5S rRNA as shown in Fig 3B with an even higher correlation between predicted and actual ASA values (*r* = 0.712). These results are consistent with larger non-redundant datasets for cross validation (89 RNA chains) and independent test (44 RNA chains) in the original RNAsnap method paper [39] and, thus, provided the confidence for our intended analysis on temperature adaptation of solvent accessibility of rRNA and tRNA. Compared to natural sequences (tRNA or rRNA in Fig 3A and 3B), predicted ASA values of a single randomly shuffled sequence are mostly featureless, indicating that all RNA bases have a similar level of exposure to solvent, and, thus, are flexible because a rigid structure would have some residues buried and other exposed (larger fluctuation). In other words, RNAsnap can distinguish a random sequence from a natural tRNA/rRNA sequence.

### Predicted and actual ASA values for mRNA

This 5IB8 structure complex also captures a segment of 30-base *E*. *coli*’s mRNA in translation. As shown in Fig 3C, the mRNA is in its open but fixed conformation through its binding to the ribosomal machinery. By comparison, predicted ASA values of the natural mRNA sequence are essentially the same as those of the corresponding random sequence, confirming that this mRNA is structureless, flexible coil, when not binding to the ribosomal machinery. This agreement between the mRNA structure in ribosome complex and the predicted solvent accessibility may be coincidental because the mRNA here has to open for translation and some mRNAs are predicted to have tertiary structures as shown below. Unfortunately, mRNA structures are only determined in ribosome complexes. There are no other mRNA structures available for comparison.

### Solvent exposure in temperature adaptation

**Fig 4:**
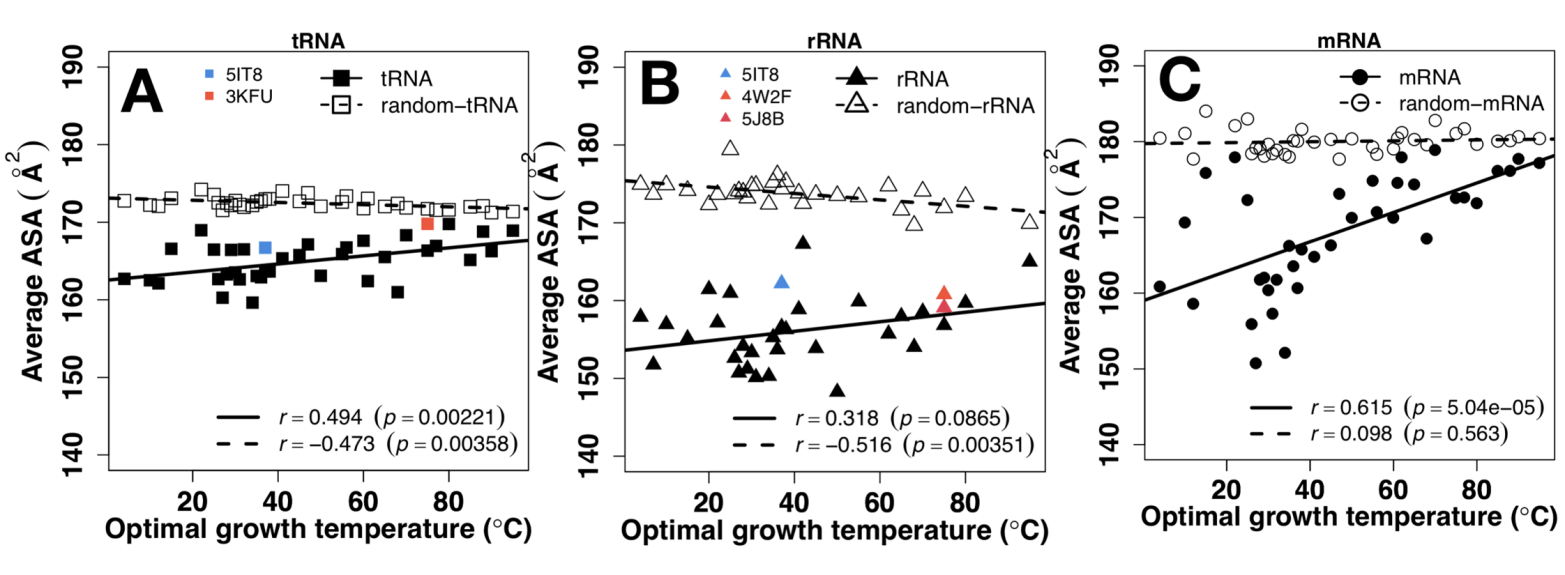
Tertiary structure vs. temperature. The average solvent accessible surface area (ASA) by RNAsnap as a function of optimal growth temperature for tRNA (A), rRNA (B), and mRNA (C). Results from random sequences are shown in open symbols. Colored points are results directly calculated from known tRNA and rRNA structures as labeled.

The overall exposure (the average ASA) of a RNA chain reflected its overall packing. More exposed chains are less compact or more extended (i.e. potentially less structured and more flexible). Fig 4 shows that all RNA chains (tRNA, rRNA, and mRNA) have much lower average exposure than corresponding random sequences, indicating that all RNA chains are less accessible (i.e. more compact) than random sequences. As a comparison, experimentally measured ASA values (colored points) from a few known tRNA and rRNA structures are plotted along with predicted values. Computational and experimental values are in the same range and similar trend. The average ASA values of tRNA, rRNA, and mRNA positively correlate to OGT (*r* = 0.494, 0.318, 0.615, respectively) and approach to values of random sequences. However, changes of average ASA values in tRNA (from 162 to 168Å^2^) and rRNA (from 150 to 160Å^2^) are much smaller than those in mRNA (from 160 to 180Å^2^) as OGT changes from 0 to 100°C, reflecting the maintenance of tRNA and rRNA but not mRNA structures as temperature increases.

**Fig 5:**
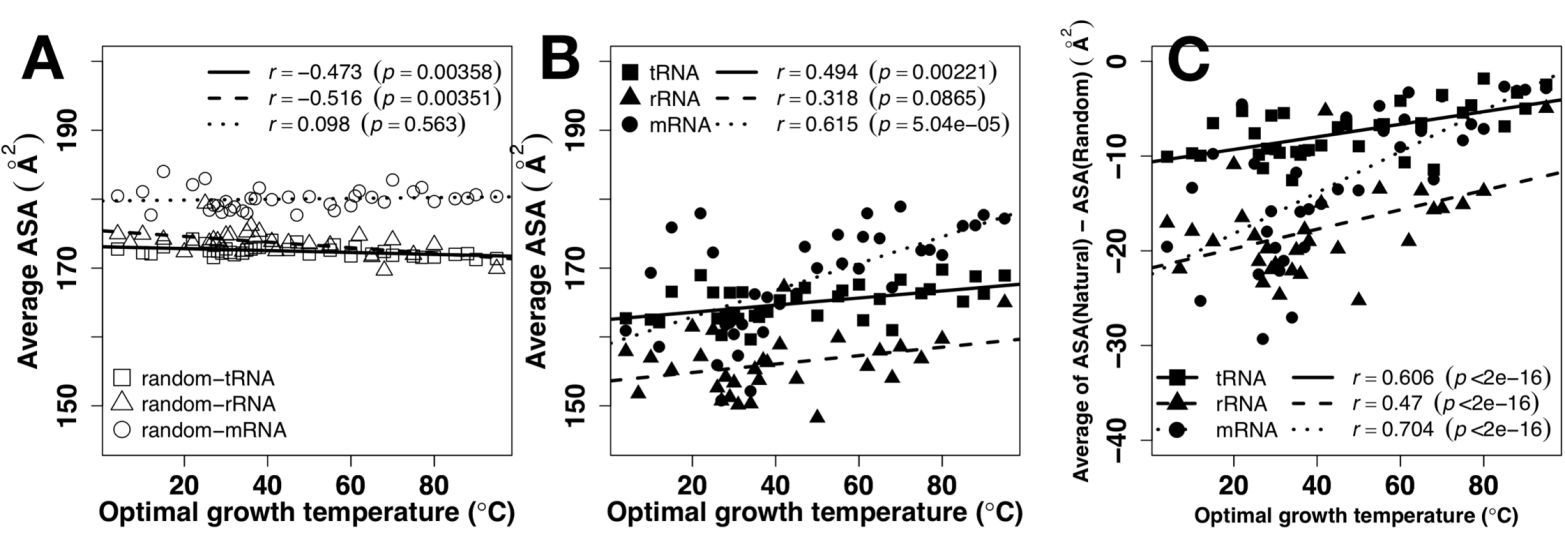
Direct comparison of ASA for different types of RNA. The average solvent accessible surface area (ASA) by RNAsnap as a function of optimal growth temperature for random sequences (A) and natural sequences (B). The relation between ASA and temperature after removing the reference ASA from random sequences is shown in (C).

What is particularly revealing is when ASAs of tRNA, rRNA and mRNA are compared in the same figure for random (Fig 5A) and actual sequences (Fig 5B). The average ASA values of random sequences are lower for tRNA and rRNA and higher for mRNA. This indicates that the compositions of structural RNAs (tRNA and rRNA) are selected to be less solvent accessible. The differences are statistically significant (p<2.2e-16 between tRNA and mRNA and 2.4e-16 between rRNA and mRNA and p = 1.6e-05 between tRNA and rRNA). Fig 5B further shows that not only compositions but also sequences were selected for structured rRNA in order to achieve stable rRNA structures at all OGTs. tRNA remains less solvent accessible than mRNA at high OGT but this is largely due to selections in nucleotide compositions as both approach to values of random sequences (Fig 4A and 4C). To remove compositional bias, we subtracted ASA of random sequences from ASA of natural sequences. As shown in Fig 5C, the correlation coefficients between ASA and OGT increases from 0.494 to 0.606 for tRNA, 0.318 to 0.470 for rRNA, and 0.615 to 0.704 for mRNA. In other words, all RNAs increase exposure to solvent as OGT increases with fastest increase in mRNA.

**Fig 6:**
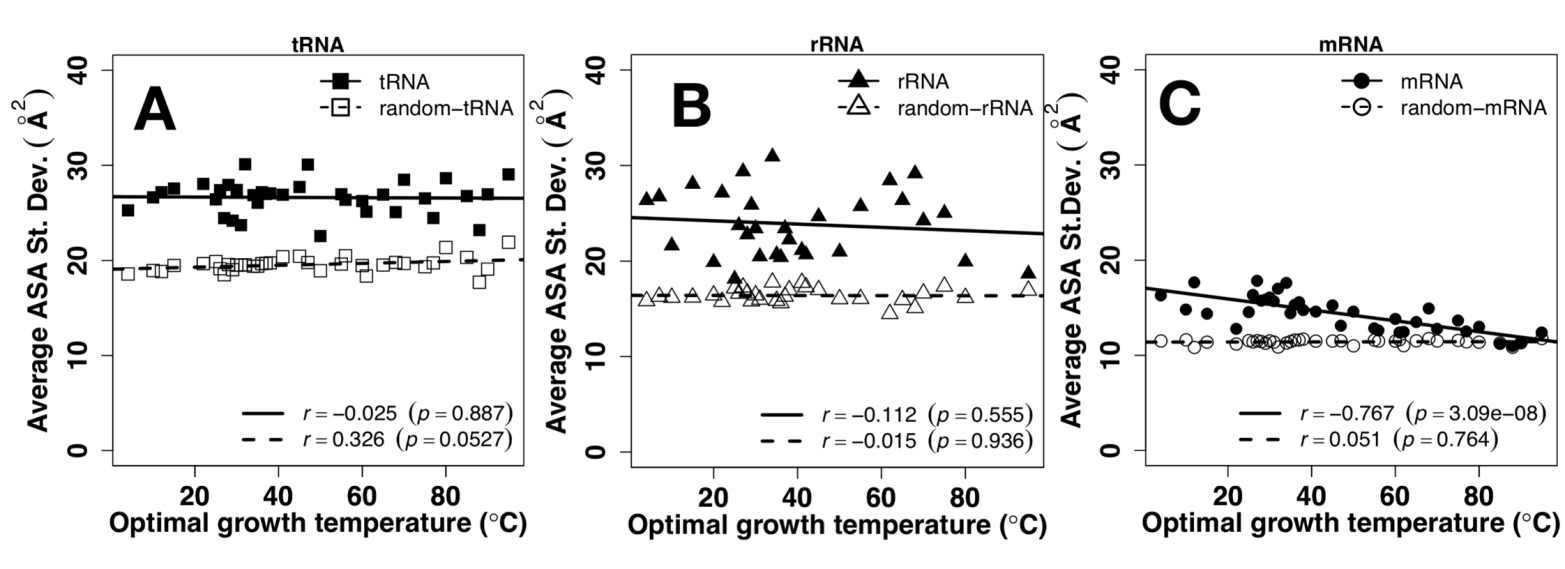
ASA fluctuation vs. Temperature. The standard deviation of solvent accessible surface area (ASA) by RNAsnap as a function of optimal growth temperature for tRNA (A), rRNA (B), and mRNA (C). Results from random sequences are shown in open symbols.

**Fig 7:**
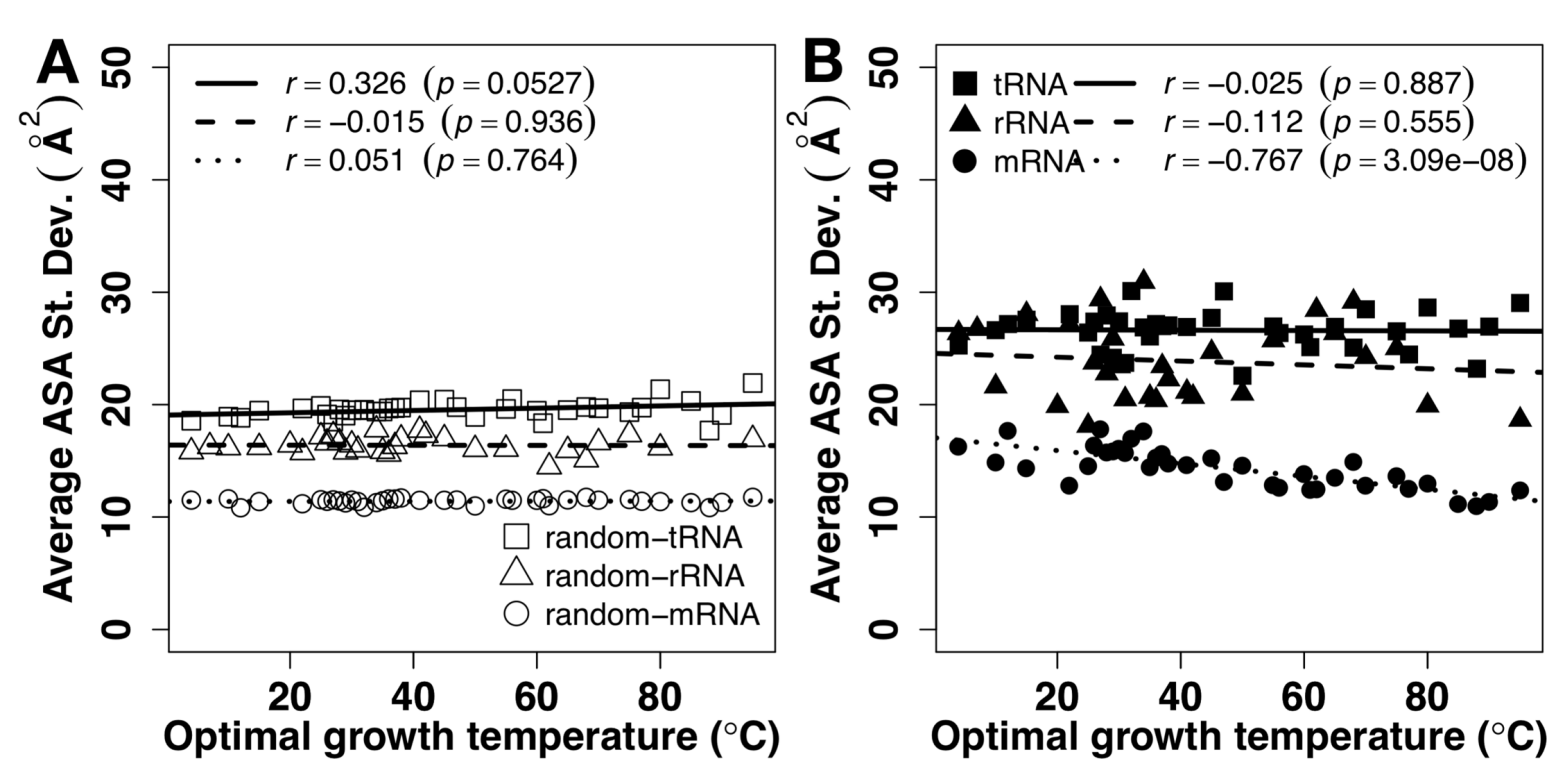
Direct comparison of ASA fluctuation for different types of RNA. The average of standard deviation of solvent accessible surface area (ASA) by RNAsnap as a function of optimal growth temperature for random sequences (A) and natural sequences (B).

The magnitude of fluctuation of ASA can be described by standard deviation of ASA values in each RNA chain. It indicates how much the ASA of the nucleotides within an RNA molecule differs from the average ASA of the entire RNA molecule. Standard deviations of ASA can be used to indicate if an RNA is fully flexible or can fold into a well-defined tertiary structure because structured RNAs will have a relatively wide distribution of solvent accessibility ranging from deeply buried, partly exposed to fully exposed nucleotides whereas in a flexible RNA, each nucleotide will be as nearly equally exposed as others due to dynamic motion. Indeed, as shown in Fig 1A and 1B, ASA values of random sequences are mostly flat and featureless, compared to structured tRNA and rRNA. As shown in Fig 6, the average standard deviation of the ASA for tRNA and rRNA are much higher than that of mRNA, consistent with the fact that tRNA and rRNA fold into defined tertiary structures for their function. By comparison, all random sequences have significantly lower standard deviations. The lack of dependence of the average standard deviation of ASA on the OGT of tRNA and rRNA (nearly flat regression line) indicates that their structures of tRNA and rRNA persist at high OGT. By contrast, the average standard deviation of ASA for mRNA is negatively correlated with the OGT (*r* = -0.767, p = 3.086e-08), approaching to nearly constant but really low standard deviation for random mRNA sequences, indicating fully flexible mRNA structures at high OGT. Fig 7 compares fluctuations of ASA values of mRNA, tRNA and rRNA directly in the same figure for random (Fig 7A) and actual (Fig 7B) sequences. Standard deviations of mRNA ASA are much lower than those of tRNA and rRNA for both random and natural sequences, confirming prewiring of mRNA sequences for flexibility, regardless of OGT.

## Discussion

In this paper, we investigated the dependence of primary, secondary, and tertiary structures (solvent accessible surface area) of structural (tRNA and rRNA) and informational (mRNA) RNAs on OGT. The newly developed program RNAsnap provides an opportunity to examine how RNA sequences code RNA structures differently for species with different OGTs. Different temperature adaptation schemes are observed.

The observed role of RNA tertiary structures in temperature adaptation relies heavily on the accuracy of the ASA predictor RNAsnap. We demonstrated its accuracy by applying it to a newly solved crystal structure containing 5S rRNA, Lysine-tRNA structures and a mRNA segment (Fig 3). The correlation coefficients between predicted and actual ASA of RNAs are 0.6 and 0.7, respectively, consistent with the reported accuracy using larger cross-validation and independent test sets [39]. Lack of structures for single random sequences of mRNA, rRNA, and tRNA (high exposure and low fluctuation) are consistent with our expectation for random sequences. Moreover, rRNA and tRNA are more structured (low exposure, high fluctuation) than mRNA, consistent with their respective main functional roles. Although not every base has an accurately predicted ASA, the average trends observed for tRNA, rRNA, and mRNA are likely real because all RNA sequences would be subjected to the same systematic errors whereas random errors would cancel each other during average. In fact, available experimental data of ASA values for tRNA and rRNA are consistent with computational trends (Fig 4A and 4B).

Sequences of tRNA and rRNA are prewired for structures not only at the sequence level but also at its composition level. tRNA and rRNA have higher GC contents than mRNA (Fig 1). Both have a positive correlation between their GC contents and OGT (Fig 1A and 1B), consistent with previous studies [19, 24–26]. There is a small increment of secondary structure of tRNA at higher OGT but this increment is largely contributed by similar increment observed for its random sequence (Fig 2A). In other words, increment of secondary structure contents are largely controlled by GC contents. For rRNA, secondary structure contents (Fig 2B) were optimized against high OGT because the behavior of natural sequences is different from that of random sequences. Prewired compositional bias of tRNA and rRNA sequences toward structural folding is further demonstrated by significantly lower average but much higher fluctuation of ASA values of random tRNA and rRNA sequences than those of mRNA. Higher fluctuation indicates the formation of a well-defined structure with large ASA difference between deeply buried and largely exposed nucleotides. Although both tRNA and rRNA increase their solvent exposure at high OGT, their fluctuations are mostly flat, relative to changes in OGT, suggesting maintenance of overall structures despite slight increase in overall solvent exposure likely due to stronger dynamic motions at high OGT. The above results of subtle difference in structural preference may be interpreted by the difference in respective functions of rRNA and tRNA. tRNAs bind amino acids and transfer them to the ribosome whereas rRNAs are ribozymes that catalyze the peptide-bond formation to construct proteins. Enzymes catalyze chemical reactions by employing rigid structures to stabilize reaction transition states while binding interactions can involve with more flexible structures. In other words, rRNA likely requires more stable structures than tRNA in order to function, which is consistent with what is observed in Figs 4 and 5.

mRNA, on the other hand, is prewired for flexibility at compositional and sequence levels. Consistent with previous studies [19, 24, 28], there is no correlation between GC contents and OGT (Fig 1C). Their secondary structure show no statistically differences between random and natural sequences (Fig 2C). There is a compositional bias toward less secondary structure content with lower stability in terms of MFE at high OGT. Random sequences of mRNA have much higher average and low fluctuation of ASA values than those of tRNA and rRNA, indicating that the composition of mRNA sequences was biased toward flexibility without structures. For low-OGT species, mRNA solvent exposures of natural sequences are much lower than their random sequences (Fig 4C) and similar to those of rRNA and tRNA (Fig 5B), indicating the existence of some tertiary structure contents. However, these mRNA structures are unlikely as well defined as those of tRNA and rRNA because the fluctuation of ASA values of mRNA remains smaller than those of tRNA and rRNA (Fig 7A). For high-OGT species, the average and fluctuation of mRNA approaches to those of random sequences (Figs 4C and 6C), indicating fully flexible mRNA conformations. These results suggest that mRNA in hyperthermophiles acts as information carriers only. However, some mRNA conformations of mesophiles and psychrophiles have well-defined tertiary structures based on their average values and fluctuation of solvent exposure, potentially with new moonlighting roles of interacting with regulatory proteins. In human cells, *in vivo* experimentally measured accessibility of mRNA to dimethyl sulfate (DMS) is similar to those structured RNAs [40] and these mRNA sequences interact with at least 860 RNA-binding proteins [54, 55]. Having tertiary structures for mRNA in mesophiles and psychrophiles but not in thermophiles could be interpreted as follows. The main function of mRNA is to carry protein-coding information and its tertiary structure is used for optional “moonlight” functions that were likely gained when evolved to live at friendlier low temperature after life was emerged from hostile high-temperature environment [56].
